# Beyond the Obvious: The Missing Pieces in Hypertriglyceridemia-Induced Pancreatitis

**DOI:** 10.7759/cureus.72140

**Published:** 2024-10-22

**Authors:** Muhammad A Zaman, Karly Milton, Mina Jilani

**Affiliations:** 1 Internal Medicine, Conemaugh Memorial Medical Center, Johnstown, USA; 2 Emergency Medicine, Lake Erie College of Osteopathic Medicine, Pittsburg, USA; 3 Internal Medicine, Services Hospital Lahore, Lahore, PAK

**Keywords:** acute pancreatitis, familial chylomicronemia syndrome, hypertri, hyper-triglyceridemia, icosapent ethyl, metabolic syndrome (mets)

## Abstract

The incidence of acute pancreatitis in the United States has been increasing for the last few years and hypertriglyceridemia is a common cause of acute pancreatitis. In this case report, we present an interesting case of a young patient with hypertriglyceridemia-induced acute pancreatitis. The patient had multiple causes for increased blood triglyceride levels, including recently started icosapent ethyl, metabolic syndrome, and possible familial chylomicronemia syndrome.

## Introduction

According to The American Gastroenterological Association, the incidence of acute pancreatitis ranges from five to 30 cases per 100,000 individuals, with evidence suggesting a rising trend. It is a leading cause of inpatient care among gastrointestinal conditions in the United States, with more than 275,000 annual hospitalizations, costing approximately $2.6 billion per year [1]. Other than gallbladder stones and alcohol, which make up 80% of acute pancreatitis cases, hypertriglyceridemia is the third most common cause, accounting for 10% of all acute pancreatitis episodes. The causes of hypertriglyceridemia are multifaceted. The genetic causes include familial chylomicronemia syndrome (FCS) and environmental factors, including lifestyle changes, medical conditions like metabolic syndrome (MetS), and diet. Hypertriglyceridemia is highly prevalent in MetS patients, with studies indicating that it is present in approximately 74%-79.6% of such individuals [2,3]. Triglyceride levels ≥150 mg/dL are the second most common component of MetS. In this case report, we will address severely elevated hypertriglyceridemia-induced pancreatitis in a 25-year-old male with metabolic syndrome who was recently started on icosapent ethyl.

## Case presentation

Presentation

A 25-year-old male presented to the emergency department emergency room with generalized abdominal discomfort for five days. On arrival to ED, his vitals were: blood pressure 156/102 mm Hg, respiratory rate 17 per minute, heart rate 79 beats per minute, temperature 96.6 F (35.9 ºC), and SpO2 96% on room air. The medical history is significant for insulin-dependent type two diabetes mellitus, hypertension, dyslipidemia, and class II obesity, with a basal metabolic index of 36.5. The patient was currently taking amlodipine 5 milligrams (mg) twice daily, atorvastatin 80 mg once daily, fenofibrate 160 mg once daily, carvedilol 6.25 mg twice daily, long-acting insulin, pre-meal short-acting insulin, omega-3 fatty acids-fish oil, and vitamin E supplements one tablet daily. The patient had also recently been prescribed icosapent ethyl 2 grams twice daily eight weeks ago by his endocrinologist after the first episode of acute pancreatitis in an attempt to control his hypertriglyceridemia better. The patient has complied with all his medications and denied any medication changes in the past few months. 

Initial diagnostic workup

**Figure 1:**
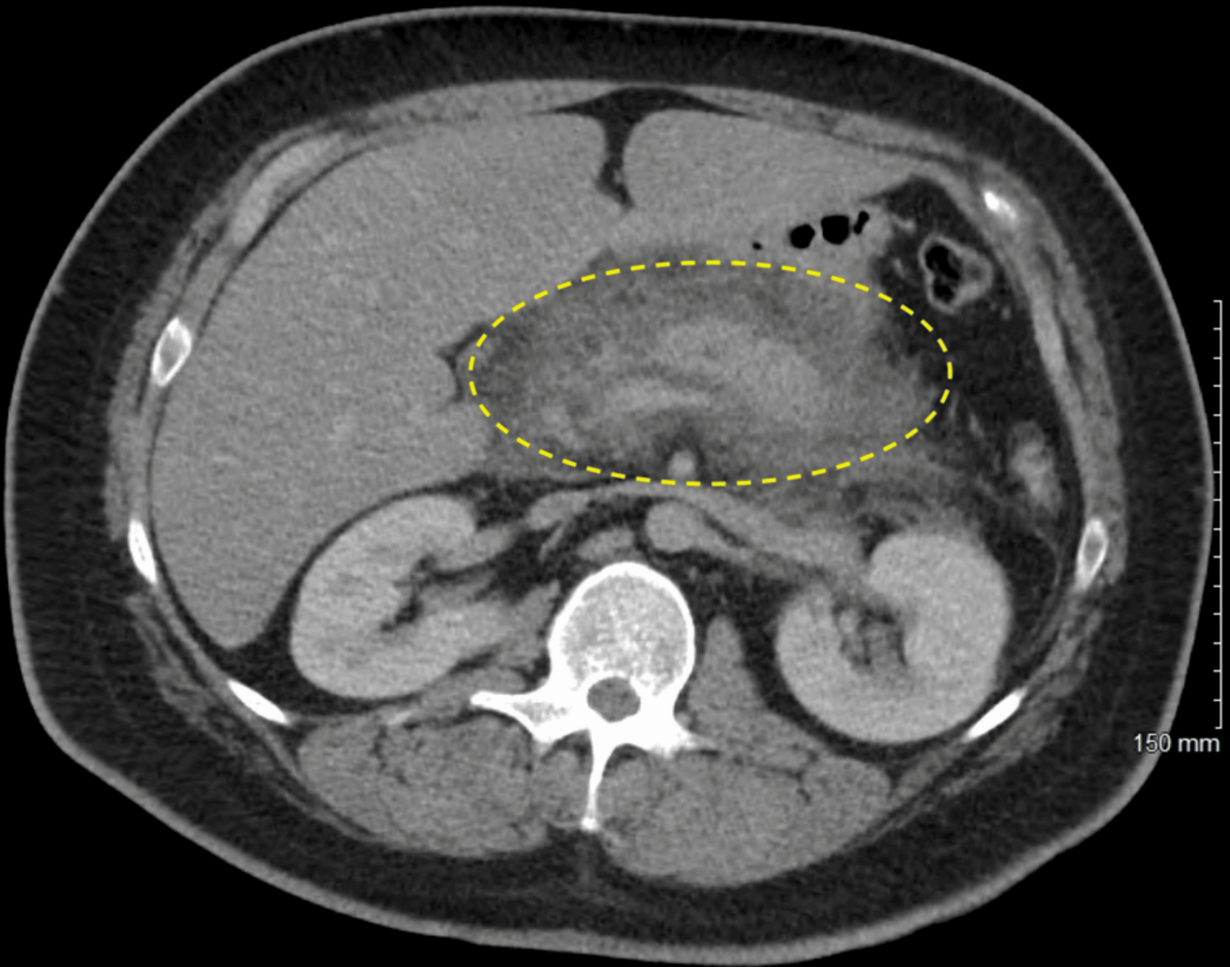
Axial section of computed tomography reveals pancreatitis and peri-pancreatic inflammation

**Figure 2:**
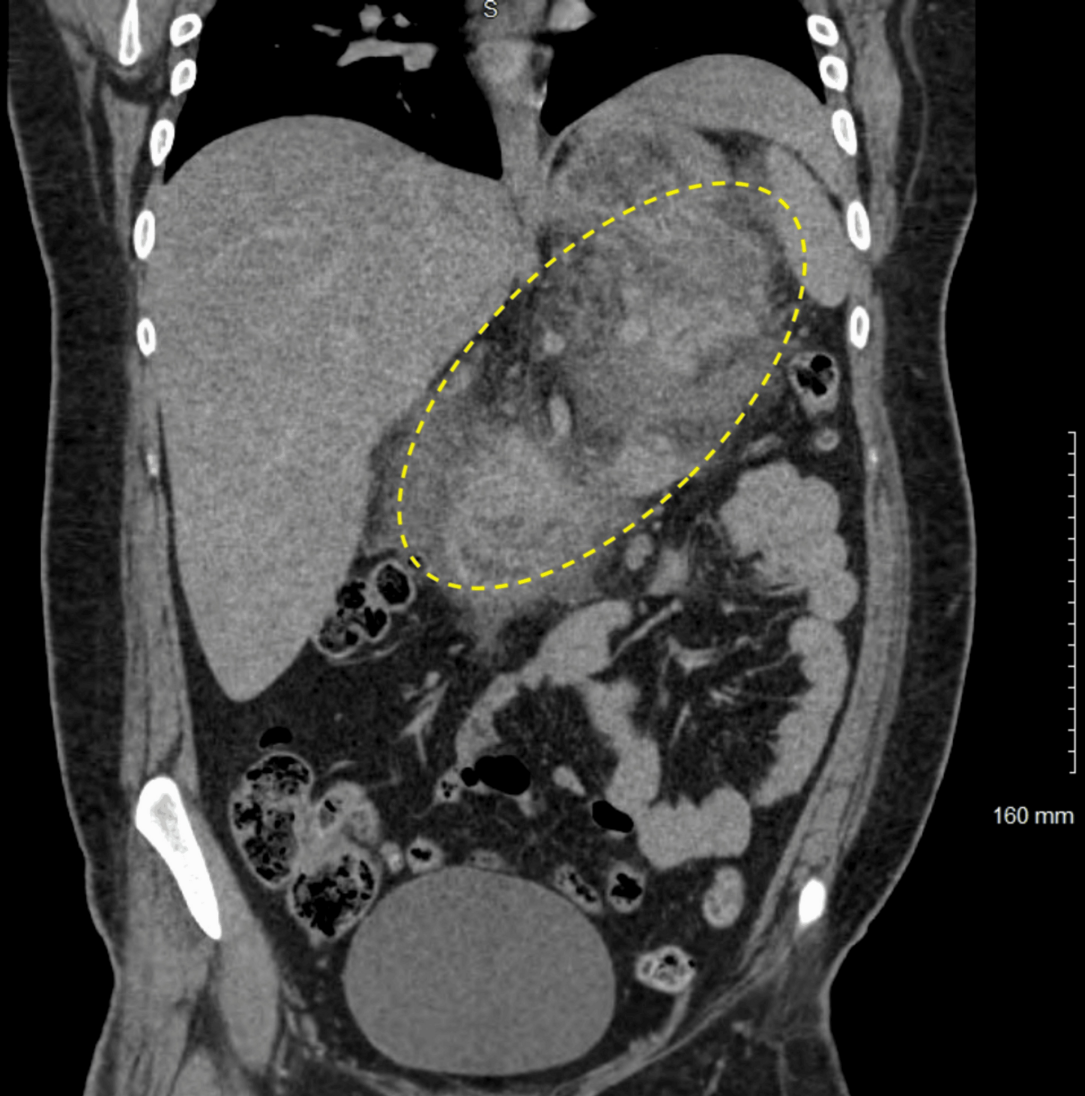
Coronal section of computed tomography reveals pancreatitis and peri-pancreatic inflammation

The initial blood draw was significant for a white opaque serum and could not be centrifuged enough for analysis. The laboratory team had to change the centrifugal machine to a super analyzer. His initial labs are significant for significantly elevated triglycerides of greater than 10,000 mg/dL. The serum lipase was 1,273 U/L. Table 1 demonstrates the detailed blood work on the initial presentation. The urine drug screen was unremarkable for any potential cause of acute pancreatitis. Urinalysis was significant for glycosuria and moderate ketonuria. Computed tomography of the abdomen and pelvis revealed a poorly defined and mildly enlarged pancreas with severe surrounding peripancreatic edema and inflammation, edema, and fluid consistent with severe acute pancreatitis. There was no evidence of gallbladder stones, which was further confirmed by bedside gallbladder ultrasound. However, there was no definitive pancreatic necrosis, pseudocyst, or abscess collection on initial imaging. Figures 1, 2 reveal the axial and coronal pancreas sections, respectively, on computed tomography scans. 

**Table 1:** Initial laboratory workup at presentation *Hemoglobin was obtained from a saline replacement procedure from a grossly lipemic blood specimen.

Laboratory test	Reference value	Patient’s test results	Laboratory test	Reference value	Patient's test results
White cell count	3.10 – 8.50 10*3/µL	14.7	BUN	8 – 26 mg/dL	19
Red blood cell count	4.50 – 6.30 10*6/µL	3.9	Creatinine	0.70 – 1.30 mg/dL	1.5
Hemoglobin	14.0 – 18.0 g/dL	14.2*	Serum glucose	83 – 110 mg/dL	336
Hematocrit	40% – 54%	33	Beta-hydroxybutyrate	0.02 – 0.27 mmol/L	3.01
Neutrophils	38.0% – 70.0%	65	Lipase	12 – 53 U/L	1,273
Lymphocytes	20.0% – 40.0%	28	Amylase	25 – 125 U/L	520
Bands	0% – 5%	2	Cholesterol	0 – 200 mg/dL	567
Sodium	136 – 145 mmol/L	127	Triglycerides	≤ 150 mg/dL	>10,000
Potassium	3.5 – 5.1 mmol/L	5.5	High density lipoproteins	≥ 55 mg/dL	9.3
Chloride	98 – 107 mmol/L	101	Low density lipoproteins	0 – 100 mg/dL	108
Bicarbonate	22 – 31 meq/L	12	Lactate	0.5 – 2.0 mmol/L	2.7
Calcium	8.50 – 10.30 mg/dL	5.8	Total bilirubin	0.3 – 1.2 mg/dL	0.3
Magnesium	1.6 – 2.6 mg/dL	1.6	Alanine transferase	0 – 55 U/L	27
Troponin I high sensitivity	0.00 – 75.00 ng/L	< 3	Alkaline phosphatase	40 – 150 mg/dL	63

Management

**Figure 3:**
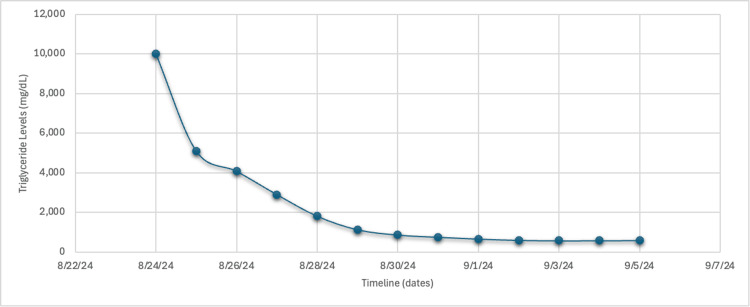
Trend of triglyceride levels during hospitalization

The patient was started on a continuous infusion of 5% dextrose at 150 mL/hr and insulin at 0.1 units/kg/hr. His condition significantly improved with insulin therapy alone, and plasmapheresis was not needed. Figure 3 shows the downtrend of triglyceride levels.

Follow up

After eight days of hospitalization, the patient was discharged home for further outpatient workup. Unfortunately, the patient lost follow-up after discharge from the hospital. The reason for the loss of follow-up was not evident.

## Discussion

Although hypertriglyceridemia is defined as any serum triglyceride level over 150 mg/dL, only very intense elevations in serum triglycerides, mostly more significant than 1,000 mg/dL, are harmful enough to precipitate acute pancreatitis. Patients with severe hypertriglyceridemia have a 14% likelihood of acute pancreatitis in their lifetime. Hypertriglyceridemia is also the leading cause of acute pancreatitis in pregnancy, seen in over 56% of cases [3]. Also, hypertriglyceridemia-induced pancreatitis must be considered in patients who have recently started on new medication and even patients with concerns of drug abuse in relevant clinical settings [4,5] on newly started medications and MetS. Patients with a primary disorder such as FCS are more often associated with severely elevated triglycerides compared to those with secondary hypertriglyceridemia from diabetes, medications, or lifestyle choices. 

Although the complete pathophysiology of hypertriglyceridemia in acute pancreatitis is not entirely clear, it is postulated that the hydrolysis of triglycerides into free fatty acids (FFA) via pancreatic lipase may induce toxic inflammatory effects. These FFA activate pro-inflammatory pathways by activating toll-like receptors 2 and 4. The Lipotoxic damage associated with elevated lipase activity has also been shown to increase intracellular calcium release, inhibit mitochondrial complexes I and V, and induce pancreatic acinar necrosis. The FFA can also contribute to the damage of various other organs, causing associated renal tubular toxicity and acute respiratory distress syndrome [6]. 

In addition to pain management and stabilization of electrolyte abnormalities, treatment of acute pancreatitis secondary to hypertriglyceridemia should focus on intravenous fluids and insulin. The insulin slows lipolysis and reduces endogenous triglyceride production in the liver [7]. In extreme situations, including pregnancy, therapeutic plasma exchange (TPE) may be utilized to lower triglyceride levels rapidly. Once recovered and stabilized, patients should be counseled about lifestyle modifications and a healthy diet and should be started on lipid-lowering medications. Generally, serum triglycerides should be kept under 500 mg/dL to prevent a recurrent episode of acute pancreatitis, though the ideal level remains under 150 mg/dL. The ability to quickly lower triglyceride levels in pancreatitis patients has yet to help improve outcomes and prevent the development of serious complications, as lipotoxic damage has already occurred in most instances. It further highlights the importance of primary prevention of hypertriglyceridemia and early screening of inherited dyslipidemia in the patients at risk. 

Familial chylomicronemia syndrome

The frequency of FCS, a rare autosomal recessive disorder characterized by severe hypertriglyceridemia, is estimated to be approximately one in one million. This rarity underscores the importance of accurate diagnosis and differentiation from more common forms of hypertriglyceridemia, such as multifactorial chylomicronemia syndrome (MCS), which has a higher prevalence of around one in 600 [8]. Other than clinical presentation, including severely elevated triglyceride levels, recurrent pancreatitis, and xanthomas, the diagnosis of FCS relies on genetic testing, lipoprotein lipase activity, and a recently validated FCS clinical scoring system. Genetic testing, the gold standard diagnosis, involves identifying bi-allelic loss of function mutations in *LPL* and *APOC2* genes. These both contribute to the function of lipoprotein lipase and its ability to metabolize chylomicrons. 

The FCS score was first introduced by Moulin et al. in 2018, a recent ethnically diverse cohort from the United Kingdom FCS Registry study by Bashir et al. The FCS score demonstrated a sensitivity of 96% and a specificity of 75% at the recommended threshold of ≥10 points [9,10]. It is also helpful in distinguishing FCS from MCS. Another testing, like post-heparin plasma LPL activity, is indicative of FCS. The FCS score was 11 for our patient (Table 2). 

**Table 2:** Familial chylomicronemia score FCS; familial chylomicronemia syndrome, TG; Triglycerides ^a^Plasma triglyceride concentrations measured at least one month apart. ^b^Secondary factors include alcohol, diabetes, metabolic syndrome, hypothyroidism, corticotherapy, and additional drugs. ^c^If the diagnosis is made during pregnancy, a second assessment is necessary to confirm diagnosis post-partum.

Moulin FCS score	Scores attributed	Score in our case
1. Fasting TG > 10mmol/L for 3 consecutive blood analyses ^a^	+5	+5
Fasting TGs > 20 mmol/L at least once	+1	+1
2. Fasting TG < 2 mmol/L	-5	0
3. No secondary factors ^b^ (expect pregnancy ^c^ and ethinyl estradiol)	+2	0
4. History of pancreatitis	+1	+1
5. Unexplained recurrent abdominal pain	+1	+1
6. No history of familial combined hyperlipidemia	+1	+1
7. No response (TG decrease < 20%) to hypolipidemia treatment	+1	+1
8. Onset of symptoms at age:		
Less than 40 years	+1	+1
Less than 20 years	+2	0
Less than 10 years	+3	0
Total score		+11

This validation study identified additional predictors such as non-European ethnicity, parental consanguinity, BMI < 25 kg/m², and recurrent pancreatitis. However, these extra factors did not significantly improve the diagnostic performance of the standard FCS score. In the clinical context, the FCS score is primarily a clinical tool and may not replace the need for genetic testing, which remains the gold standard for diagnosis. The score should be used as an adjunct to, rather than a replacement for, comprehensive clinical and genetic evaluation.

The newer treatment options for FCS include apolipoprotein C-III inhibitors (Volanesorsen), angiopoietin-like protein three inhibitors (Evinacumab), microsomal triglyceride transfer protein inhibitors (Lomitapide), and gene therapy targeting LPL [11-13]. These novel therapies represent significant advancements in managing FCS, offering hope for better control of triglyceride levels and reduced pancreatitis risk.

Metabolic syndrome (MetS)

Approximately 31% of the adult population in the United States has an elevated triglyceride level of over 150 mg/dL. About 1%-2% of this subset had levels categorized as “very high” or above 500 mg/dL [14]. There is a direct association between MetS and acute pancreatitis. A case-control study by Shen et al. found that the incidence rate of MetS in acute pancreatitis patients was significantly higher than in controls, with an odds ratio (OR) of 2.837 [15]. Further studies using Mendelian randomization approaches have also confirmed a genetic and causal relationship between MetS and acute pancreatitis [16]. Another study by Mikolasevic et al., studying 609 patients, found that patients with MetS had a higher incidence of moderately severe and severe acute pancreatitis, as well as higher mortality rates compared to those without MetS [17]. The patient in our case report also has MetS. However, the patient was compliant with medication and was losing weight at the time of the severe acute pancreatitis episode. Although not impossible, an acute rise from 465 mg/dL to greater than 10,000 mg/dL triglycerides cannot be attributed to MetS alone, especially with good medication compliance.

Icosapent ethyl

Icosapent ethyl is a relatively new medication primarily used to lower triglyceride levels in patients with severe hypertriglyceridemia. Real-world registry data and primary hypertriglyceridemia trials have demonstrated Icosapent ethyl’s efficacy [18]. However, there have been isolated reports of increased blood triglyceride levels as an adverse reaction during FDA post-marketing surveillance. In our report, the patient was started on Icosapent ethyl six to eight weeks ago. The calculated naranjo score for adverse drug reactions for Icosapent ethyl was 3, suggestive of possible drug-related adverse reactions. Although these reports do not outweigh the substantial evidence supporting the triglyceride-lowering effects of Icosapent ethyl, it is safe practice to keep in mind such possibilities. Of note, the impact of Icosapent ethyl on the risk of pancreatitis in patients with severe hypertriglyceridemia has not been determined [19].

## Conclusions

Physicians should be aware of identifying its etiology simultaneously while treating acute pancreatitis and treating patients with hypertriglyceridemia-induced pancreatitis. The recently validated FCS score can be used as a good screening tool and is easy to perform. However, genetic studies remain the gold standard of diagnosis. Other than isolated reports of elevated blood triglycerides by icosapent ethyl, it has an established safety profile. Clear evidence of an association between hypertriglyceridemia-induced pancreatitis and icosapent ethyl is lacking.

## References

[1] Peery AF, Crockett SD, Barritt AS (2015). Burden of gastrointestinal, liver, and pancreatic diseases in the United States. Gastroenterology.

[2] Shojaee-Moradie F, Ma Y, Lou S, Hovorka R, Umpleby AM (2014). Prandial hypertriglyceridemia in metabolic syndrome is due to an overproduction of both chylomicron and VLDL triacylglycerol. Diabetes.

[3] Chang CC, Hsieh YY, Tsai HD (1998). Acute pancreatitis in pregnancy. Chin Med J.

[4] Goraya MH, Abbasi EU, Amin MK (2023). Acute pancreatitis secondary to tamoxifen-associated hypertriglyceridemia: a clinical update. J Oncol Pharm Pract.

[5] Goraya MH, Malik A, Inayat F, Ishtiaq R, Zaman MA, Arslan HM, Tarar ZI (2021). Acute pancreatitis secondary to cocaine use: a case-based systematic literature review. Clin J Gastroenterol.

[6] Wan J, He W, Zhu Y (2017). Stratified analysis and clinical significance of elevated serum triglyceride levels in early acute pancreatitis: a retrospective study. Lipids Health Dis.

[7] Ewald N, Hardt PD, Kloer HU (2009). Severe hypertriglyceridemia and pancreatitis: presentation and management. Curr Opin Lipidol.

[8] Paragh G, Németh Á, Harangi M, Banach M, Fülöp P (2022). Causes, clinical findings and therapeutic options in chylomicronemia syndrome, a special form of hypertriglyceridemia. Lipids Health Dis.

[9] Bashir B, Kwok S, Wierzbicki AS (2024). Validation of the familial chylomicronaemia syndrome (FCS) score in an ethnically diverse cohort from UK FCS registry: Implications for diagnosis and differentiation from multifactorial chylomicronaemia syndrome (MCS). Atherosclerosis.

[10] Moulin P, Dufour R, Averna M (2018). Identification and diagnosis of patients with familial chylomicronaemia syndrome (FCS): expert panel recommendations and proposal of an "FCS score". Atherosclerosis.

[11] Chaudhry R, Viljoen A, Wierzbicki AS (2018). Pharmacological treatment options for severe hypertriglyceridemia and familial chylomicronemia syndrome. Expert Rev Clin Pharmacol.

[12] Witztum JL, Gaudet D, Freedman SD (2019). Volanesorsen and triglyceride levels in familial chylomicronemia syndrome. N Engl J Med.

[13] Cefalù AB, D'Erasmo L, Iannuzzo G (2022). Efficacy and safety of lomitapide in familial chylomicronaemia syndrome. Atherosclerosis.

[14] Miller M, Stone NJ, Ballantyne C (2011). Triglycerides and cardiovascular disease: a scientific statement from the American Heart Association. Circulation.

[15] Shen Z, Wang X, Zhen Z, Wang Y, Sun P (2021). Metabolic syndrome components and acute pancreatitis: a case-control study in China. BMC Gastroenterol.

[16] Zhu S, Ding Z (2024). Acute pancreatitis and metabolic syndrome: genetic correlations and causal associations. Endocrine.

[17] Mikolasevic I, Milic S, Orlic L (2016). Metabolic syndrome and acute pancreatitis. Eur J Intern Med.

[18] Cai H, Jia B, Fu Z, Chen B, Liu Y, Zhao S (2024). Real-world safety of icosapent ethyl: analysis based on spontaneous reports in FAERS database. Expert Opin Drug Saf.

[19] (2017). GLUCOPHAGE® (metformin hydrochloride) tablets. https://www.accessdata.fda.gov/drugsatfda_docs/label/2017/202057s019lbl.pdf.

